# Suicide Trends over Time by Occupation in Korea and Their Relationship to Economic Downturns

**DOI:** 10.3390/ijerph16112007

**Published:** 2019-06-05

**Authors:** Jin-Ha Yoon, Sun Jae Jung, Jaesung Choi, Mo-Yeol Kang

**Affiliations:** 1 Department of Preventive Medicine, Yonsei University College of Medicine, Seoul 03722, Korea; flyinyou@gmail.com (J.-H.Y.); sunjaejung@yuhs.ac (S.J.J.); 2 The Institute for Occupational Health, Yonsei University College of Medicine, Seoul 03722, Korea; 3 Department of Epidemiology, Harvard T.H. Chan School of Public Health, Boston, MA 02115, USA; 4 Department of Global Economics, Sungkyunkwan University, Seoul 03063, Korea; jaesungc@skku.edu; 5 Department of Occupational and Environmental Medicine, Seoul St. Mary’s Hospital, College of Medicine, The Catholic University of Korea, Seoul 06591, Korea

**Keywords:** suicide, occupations, economics

## Abstract

We analyzed suicide mortality by occupation using administrative data from 1993 to 2016. Methods: National death records from 1993 to 2016 of the Korea National Statistical Office (KNSO) were used. Suicidal death was taken from Korean Classification of Disease codes as intentional self-harm (X60–X84) and sequelae of intentional self-harm (Y870). Occupational groups were categorized into “Manager,” “Officer,” ”Service-Trade,” “Agricultural-Fishery-Forestry” (AFF), “Skilled Manual,” and “Unskilled Manual.” Direct standardized mortality (DSM) and standardized mortality ratio (SMR) with 95% confidence interval (95% CI) were calculated. Overall, suicide rates increased during economic downturns, especially among lower socio-economic occupation classes. Both DSM and SMR were highest in AFF, followed by Unskilled Manual, Service-Trade, Officer, Skilled Manual, and Manager categories among men, whereas women showed the highest DSM and SMR in AFF, followed by Service-Trade, Officer, Unskilled Manual, Manager, and Skilled Manual categories. The age-stratified analysis showed that age groups with increasing trends in suicide differed according to occupation and gender. In certain occupational groups, the time-point prevalence fluctuated with socio-economic background in suicidal mortality and differed by age and gender.

## 1. Introduction

Suicide is an important public health problem worldwide, and the Republic of Korea has the highest suicide rate among OECD countries. In 2013, the rate of death by suicide in Korea was 28.7 per 100,000 people, which is about 2.4 times higher than other OECD countries (OECD on average, 12.1 per 100,000 people; Italy, 5.8; United States, 12.5; and Japan, 20.9) [1]. 

Suicide is an important social and medical issue, with a complex, multi-dimensional etiology [2,3,4]. Various factors contribute to suicide, including proximate causes (such as psychiatric illness, personality, psychobiological factors) and more distal causes (such as social position, economic factors, social isolation and integration, and cultural factors) [2]. There are various paradigms that explain suicide behavior according to biological, interpersonal, sociological, and economic concepts. 

In addition, suicide rates are thought to be affected by socioeconomic environment, an assumption rooted in the work of Durkheim [5]. Many researchers from various countries have reported the impact of economic factors on suicide [2,6,7,8,9,10,11]. Previous studies on the dramatic increase in suicide mortality in Southeast Asia in the late 1990s have concluded that the rise corresponded with the Asian economic crisis in 1997–1998 [12]. Suicide has become a more common cause of death during economic downturns. Several previous studies describe an increased rate of suicide during the Global Financial Crisis (GFC) in 2008 [7,11,13].

The literature on suicide has highlighted consistent relationships between certain occupational groups and suicide [14], particularly farmers, doctors, nurses, dentists, veterinarians, pharmacists, police, military personnel, seafarers, and artists. These high-risk occupational groups exhibit common risk factors regarding suicide, such as easy access to lethal means, frequent exposure to neurotoxic chemical compounds (e.g., pesticides), social isolation at work, high psychological strain, and long working hours [4,14,15]. There is also some evidence of natural selection of high-risk people to certain occupations [4]. 

These studies strongly suggest the presence of occupational discrepancies in suicide in terms of individual socioeconomic position and social context. However, few studies have compared suicide rates by occupation on a national basis and how they have changed over time. Thus, the evidence so far does not necessarily implicate any contextual effects over time. Accordingly, this study examined differences in the incidence of suicide by occupation among economically active adults in Korea, aged 20–59, using national mortality data over 20 years (1993 through 2016).

## 2. Methods

National death records from 1993 to 2016 provided by the Korea National Statistical Office (KNSO, https://mdis.kostat.go.kr) were used for the current study. This registry covers all deaths by cause for the Korean population. Causes of death were classified by physicians according to the Korean Classification of Disease (KCD) prior to public publication by KNSO, which corresponds to the International Classification of Diseases, 10th Edition (ICD-10). The registry provides/includes demographic characteristics such as birth year, gender, and occupation as well as date of death. Occupation at time of death was reported in death record. Suicidal death was defined as intentional self-harm (X60–X84) and sequelae of intentional self-harm (Y870). To focus on working populations, we analyzed workers aged 20–59, because the general retirement age is 60 in Korea. Students, housewives, and soldiers were excluded.

The nine major categories of Korean Standard Classification of Occupations and an additional special category for soldiers in death certification were used current study. Excluding soldiers, we reclassified nine categories into six occupational groups: Manager, Officer, Service-Trade, Agricultural-Fishery-Forestry (AFF), Skilled Manual (e.g., machine operators and skilled assemblers), and Unskilled Manual.

The unemployment rate and GDP growth rate from 1993 to 2017 in KNSO was used for regression analysis with the number of suicides. The definition of the unemployed used by KNSO is “those who actively sought jobs for the past four weeks […], but did not work in the week under the survey”.

The standard reference population was defined as the total economically active population from Population Census Data from 1995 to 2015 (the Korean Census survey was conducted every 5 years). The annual number in the reference population was calculated by a generalized additive model. The references for age-specific death count were calculated from death records of KNSO [16]. Hence, the age-specific death rate was defined as the ratio of the death count to the number in the reference population.

### 2.1. Approval of the Research Protocol

We used data released from the Korea Statistical Information System (KOSIS) Statistics DB [cited 10 June 2018] (http://kosis.kr/eng), freely available to researchers. The data contain no personal identifiers. The Institutional Review Board (IRB) of the Yonsei University Health System approved the current study design (IRB number: Y-2017-0100).

### 2.2. Statistical Analysis

Linear regression analysis was conducted for each occupation and age group stratum. Beta coefficient and *p* value were estimated on the association with the number of suicides, unemployment rate (%), and GDP grow rate (%). The standardized mortality ratio (SMR) and direct standardized mortality (DSM) of suicide per 100,000 person years were calculated. Age-specific reference suicide death rates and 95% confidence intervals were also calculated using the total working population from 1993 to 2016. All statistics were conducted by the R program ver. 3.4 [17]. The SMR of suicide was calculated according to the following steps. First, the age-specific reference mortality rate was obtained from the total working population. Second, the suicide mortality rate was multiplied by each stratum population size by year, gender, and occupation group to calculate the expected number of suicide cases. Finally, the SMR of suicide was calculated as the ratio of total observed number of suicides across different age groups divided by the sum of the total numbers of expected number of suicides. The SMR was multiplied by 100. Hence, an SMR of suicide more than 100 means a higher suicide rate compared to suicide rate when adjusting age effect.

For DSM, the first step was generating age-specific occupational suicide rates for each occupational group. Second, expected suicide counts were calculated by multiplying those rates with the corresponding age stratum population size of the reference group. Finally, DSM was defined as the ratio of the sum of expected suicide counts to the total population size by year, gender, and occupation. 

## 3. Results

The total observed number of deaths by suicide was 61,937 in men and 13,623 in women during the study period from 1993 to 2016. Service-Trade workers showed the highest number of suicide in both genders (15,195 in men and 5082 in women), followed by Skilled Manual workers in men (11,438) and Officers in women (2962) (Table 1). 

As presented in Table 1, the DSM values per 100,000 of Manager, Officer, Service-Trade, AFF, Skilled Manual and Unskilled Manual workers were 13.96, 24.73, 35.37, 62.75, 14.06, and 38.54 in men and 5.83, 8.80, 9.91, 18.43, 3.44, and 7.71 in women. The SMR were higher in AFF, Unskilled Manual, Service-Trade, and Office categories in men, in that order, during the total period. For women, the SMR were higher for AFF, Service-Trade, and Officer, in that order.

**Figure 1:**
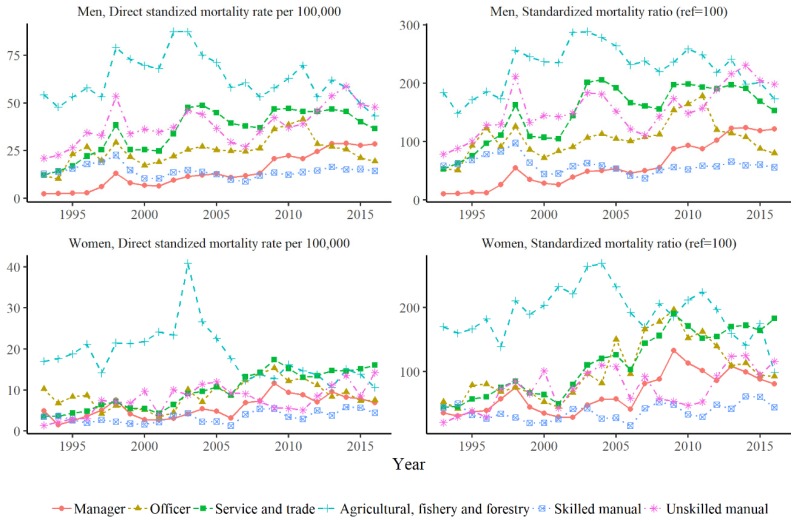
Direct standardized mortality rate per 100,000 and standardized mortality ratio (ref = 100) from 1993 to 2016. Reference population was total working population during 1993–2016.

The DSM for AFF was 54.23 at the start and 43.26 at the end, with a peak value of 75.10 in 2004. The DSM per 100,000 for Skilled Manual workers was 12.83 in 1993 and 14.34 in 2016, with a peak value of 22.65 in 1998. The DSM per 100,000 in Unskilled Manual laborers at the start, end, and peak was 21.04, 47.89, and 58.82 in 2014, respectively (Figure 1 and Table S1). 

For women, the DSM of suicide per 100,000 by category at the start and end of the study period was 4.9 and 6.91 for Manager, 10.3 and 7.53 for Officer, 3.62 and 16.07 for Service-Trade, 17.02 and 10.61 for AFF, 3.42 and 4.45 for Skilled Manual, and 1.31 and 14.26 for Unskilled Manual, respectively. The peak values of the DSM by category were 11.66 in 2009 for Manager, 15.32 in 2009 for Officer, 17.43 in 2009 for Service-Trade, 40.85 in 2003 for AFF, 5.83 in 2014 for Skilled Manual, and 14.26 in 206 for Unskilled Manual (Figure 1 and Table S1).

The SMR of suicide showed differences across occupational groups (Figure 1 and Tables S2–S3). The SMR of each occupation was higher than that of the reference group in certain years. Managers that were men experienced a higher SMR from 2013 to 2015, and those that were women did in 1993. Women officers suffered a higher SMR in 2005 and from 2007 to 2012. Men in the Service-Trade category showed a higher SMR in 1997, 1998, and from 2002 to 2016, and women showed this in 2005 and from 2007 to 2016. AFF shows a higher SMR in all years of the study period for both men and women, with recent exceptions. Skilled Manual laborers of both genders never experienced a higher SMR. Unskilled Manual workers that were men had a higher SMR from 1996 to 2006 and from 2008 to 2016 but those that were women never did (Figure 1 and Tables S2–S3).

**Figure 2:**
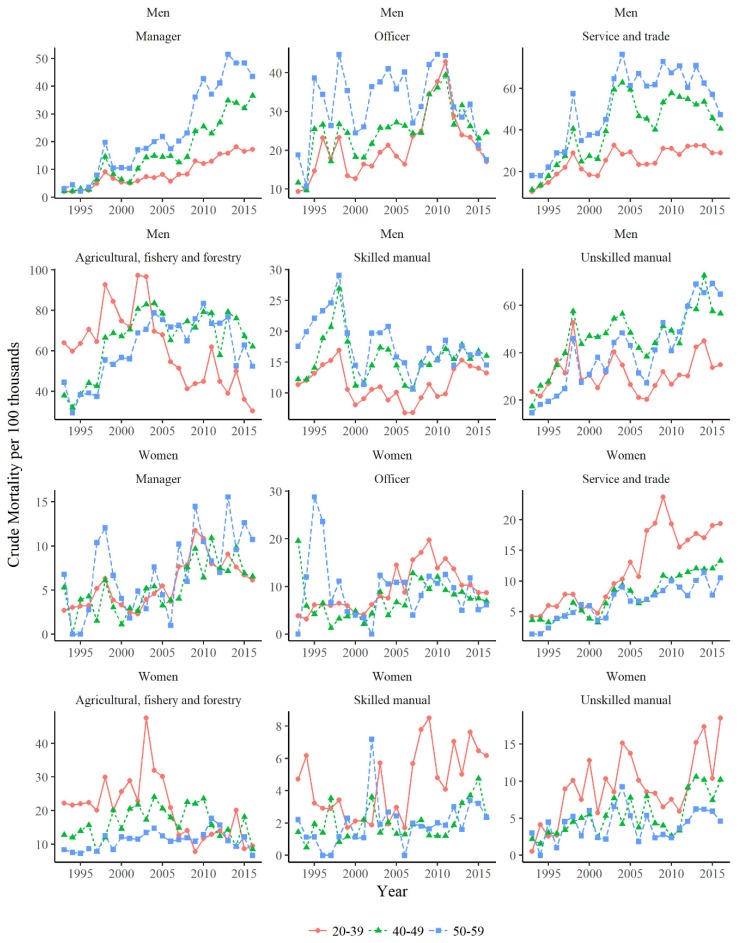
Crude mortality per 100,000 according to age groups 20–39, 40–49, and 50–59. Note: In each graph, different scales were used to highlight the comparison between age groups.

The crude suicide rate was also stratified into age groups of 20–39, 40–49, and 50–59. For men, the older age group of the Manager, Officer, Service-Trade, and Unskilled Manual categories each had higher suicide mortality. In contrast to men, younger female workers in Service-Trade, AFF, Skilled Manual, and Unskilled Manual categories had higher mortality compared to their older counterparts. In particular, Service-Trade workers showed greater gender differences: older male workers and younger female workers had a higher prevalence of suicide. Suicide prevalence in the Skilled and Unskilled Manual groups also showed a gender difference, where older male and younger female workers had a higher prevalence of suicide (Figure 2). 

Occupation and age group stratification analysis shows that the number of suicides fluctuated by GDP growth rate and unemployment rate. The 40–49 age group of Skilled Manual workers shows the largest regression coefficient (Beta = 17.800, *p* value = 0.001) on unemployment rate, the 40–49 age group of Service-Trade workers shows the largest regression coefficient (Beta = −5.11, *p* value = 0.018) on GDP grow rate (Table 2).

## 4. Discussion

In this study, we analyzed trends in occupational differences in suicide mortality in the Republic of Korea over the past two decades. Overall, there were three major peaks in suicide rate: (1) around 1998, (2) around 2003, and (3) 2008–2010. During the first and second periods, the rise in suicide rate was prominent among lower socio-economic occupation groups such as AFF, Unskilled Manual, and Service-Trade. However, there has been a steady rise in suicide rate among older male officers and managers since 2008, while suicide rate among females has continued to decline after the peak in 2009. In addition, suicides of manager, officers, and service and trade workers fluctuated with GDP growth rate, while suicides of workers in the AFF, Skilled Manual, and Unskilled Manual categories fluctuated with unemployment rate. Most vulnerable groups were middle-aged service and trade workers when the GDP growth rate declined, and middle-aged Skilled Manual workers when the unemployment rate aggravated.

Korea experienced an economic recession during the late 1990s, and the economic growth rate was −5.7% in 1998. Many researchers have reported that an increase in suicide mortality corresponded with the Asian economic crisis in 1997–1998 [12], and the most vulnerable occupations during the economic recession were those in the AFF sector [7,18]. After the economic recession of 1998, there was a rebound in economic growth, reaching around 10% in 2000. The Korean government implemented many polices to revive economic growth, including the encouragement of credit card use in the general population as a radical neoliberal restructuring of Korea [19]. The government thought that increased credit card use would encourage domestic consumption, leading to increases in employment as well as tax revenues. If the customer used a credit card instead of cash, the income of the self-employed would be exposed to the national tax service, increasing government tax revenue. Consequently, the amount of credit card usage increased 7-fold within 4 years (47 trillion Korea Won in 1998 to 330 trillion Korea Won in 2002 for banking credit cards alone), cash service accounted for 60% of the total card usage in 2002, and the number of defaulters increased almost two-fold in 2003 compared to 1997 (3.7 million in 2003) [16]. We speculate that the sharp increase in suicide from 2002 to 2003 might be due to social problems related to default on credit card debt. However, we did not analyze the amount of effect on occupational suicidal risk differences in the current study. That hypothesis should be explored after constructing a greater data set. 

Meanwhile, the third rise in suicide mortality from 2008 to 2010 may be related to the GFC. This was most apparent among older age groups of male managers (Figure 2). Managers are vulnerable to mental disorders because they are usually under a condition of specific work-related stress, with a high responsibility for their company, a lack of social support, a competitive working situation, self-blame, and sometimes low compliance with treatment. In the period of the GFC, becoming the target of corporate restructuring may have led to a severe challenge for them [20]. Moreover, persons in a manager occupation could have experienced financial loss from previous investments. In line with the findings in this study, Han et al. (2016) reported that the 12-month prevalence of suicidal ideation among employed adults in the U.S. varied by occupation based on nationally representative data in 2008–2013 [21]. They identified specific occupation groups at higher risk for suicidal ideation, including (1) media and communication workers, (2) lawyers, judges, and legal support workers, and (3) social scientists and related workers. In contrast, employees working in (1) farming, fishing, and forestry occupations, (2) engineers, architects, and surveyors, and (3) workers in food preparation occupations (machine workers) were at lower risk for suicidal ideation. However, the trend in suicidal behavior in Australia is not consistent with the above patterns. Over the period from 2001 to 2010, the major occupational groups with the highest rates of suicide related to the GFC in Australia were laborers, farmers, machine operators, and technical and trade workers [22]. The differences in results between studies might be attributed to differences across cultures, but could be also due to methodological issues, such as sample size by occupation or certification of suicide. 

Economic downturn is often accompanied by decreasing individual income, which may lead to a reduction in opportunities, as well as an elevation in individual financial burden, thereby increasing the likelihood of stressful life events [23]. In countries that have been hit hardest by the most recent recession, which started in 2007, living and working conditions have substantially worsened [9]. Working conditions became more challenging, and unemployment rates increased as a result of the downturn in the global economy and the following deterioration of the labor markets. Overall, people are more concerned about losing their jobs because of rising job insecurity. A study based on 26 European countries reported that every 1% increase in unemployment rate was associated with a 0.79% risk in suicides for age groups <65 years [24]. Levels of financial hardship and social exclusion also have worsened, especially for those who were already at risk. In addition, during economic downturns, many people may feel defeated, as if they have failed in life or in their career and that they have become a burden to others. Feelings of defeat, failure, and being a burden are all crucial features that can drive a person to suicidal behavior [25]. In particular, older male workers in Korea are more likely to have such feelings under the patriarchal Confucianism of East Asian culture.

The results of the present study suggest that trends in suicide rate across occupations differ by gender, which is in accordance with the findings of previous studies that socioeconomic differences in suicide are much larger among men than women. There were a number of other differences in suicide trends between males and females. For instance, the suicide rate decreased among older females in official and management occupations, whereas the suicide rates among males in similar occupations sharply increased. The finding of a smaller change in suicide risk for females across almost all occupational groups may suggest that Korean women of working age are less vulnerable to economic downturn compared to men. These results may be in part explained by differences in response to economic challenges between men and women. Compared to females, males react more impulsively as a result of intense feeling of economic plight [26].

The main strengths of this paper lie in the use of administrative data covering the entire Korean population for over 20 years with information on occupation. Our comparison between entire occupational groups using actual values is useful in identifying higher-risk occupational groups to develop preventative intervention programs. However, the present study also has several methodological limitations. First, occupation at the time of death was used when we calculated mortality, which may not necessarily reflect individual life-time socioeconomic position [27]. In addition, these data are based only on the given occupation classification and do not show detailed information on position within a given occupation. For example, occupations in the Service-Trade category included various jobs, such as policeman, firefighter, or retail dealer, which may vary in the risk of suicide. Second, there are concerns about the quality of cause-of-death coding and the completeness of the ascertainment of suicide cases. It is possible that the number of suicides could have been underreported because of a social bias against suicide; this may result in the presence of omitted suicide reports or false reports that described the cause of death by suicide as a traffic accident, substance intoxication, or some other cause [28]. Third, we could not fully adjust for bio-psychosocial risk factors such as history of physical and mental disorders, abuse of alcohol and other substances, and family history, environmental risk factors such as organic solvent or pesticide exposure, and socio-cultural risk factors such as a lack of social support and a sense of isolation, certain cultural and religious beliefs, and exposure to and influence by others who have died by suicide. Finally, we restricted the analysis to those who were aged 20–59 years, excluding students, housewives, military personnel, and groups with no listed occupation; therefore, our results cannot address the relationship between economic downturn and suicide mortality among those who do not belong to the prime-aged working population in the labor market.

## 5. Conclusions

The present findings demonstrate that occupational disparities in suicide rates fluctuate over time and may be exacerbated by economic downturns such as the economic crisis in the late 1990s, early 2000s, and the 2007–2009 GFC. Further, continuous increases in suicide rate for certain occupational groups that extend beyond cessation of the GFC by 2010 indicate residual impacts of the economic crisis. From a public health perspective, the findings of the present study suggest that social protection can play an important role in potential suicide prevention strategies. Our current study also shows vulnerable occupational groups, and there was age-specific heterogeneity. Occupation and age group specific strategies are needed to prevent the risk of suicide. Further investigation should focus on identifying occupational differences in mediators contributing to suicide.

## Figures and Tables

**Table 1:** Suicide and mortality ratio during 1993–2016.

Occupations	Observed Death	Direct Standardized Mortality (Per 100,000)	Standardized Mortality Ratio
Men			
Manager	8849	13.96 (13.93–13.99)	59.67 (58.43–60.93)
Officer	9515	24.73 (24.69–24.77)	108.76 (106.59–110.97)
Service-Trade	15,194	35.37 (35.32–35.42)	152.87 (150.45–155.32)
Agricultural-Fishery-Forestry	8949	62.75 (62.69–62.81)	221.55 (216.98–226.19)
Skilled Manual	11,438	14.06 (14.03–14.09)	58.99 (57.91–60.08)
Unskilled Manual	7992	38.54 (38.49–38.59)	160.18 (156.69–163.73)
Women			
Manager	2442	5.83 (5.80–5.85)	74.17 (71.26–77.18)
Officer	2962	8.80 (8.77–8.83)	110.37 (106.43–114.42)
Service-Trade	5082	9.91 (9.88–9.94)	120.38 (117.09–123.74)
Agricultural-Fishery-Forestry	1805	18.43 (18.39–18.47)	191.86 (183.11–200.92)
Skilled Manual	536	3.44 (3.42–3.46)	37.52 (34.41–40.83)
Unskilled Manual	796	7.71 (7.68–7.74)	75.38 (70.23–80.80)

Note: Reference population is total working population during 1993 to 2016.

**Table 2:** Association among unemployment rate, GDP growth rate, and the number of suicides according to occupational groups.

Age Group	Manager		Officer		Service-Trade		Agricultural-Fishery-Forestry		Skilled Manual		Unskilled Manual	
	Beta	*p*-Value	Beta	*p*-Value	Beta	*p*-Value	Beta	*p*-Value	Beta	*p*-Value	Beta	*p*-Value
Unemployment rate (%)												
20–24	−0.057	0.927	−2.213	0.065	−0.171	0.819	0.899	0.389	1.058	0.439	1.617	**0.002**
25–29	1.254	0.414	1.669	0.569	2.774	0.197	2.378	0.201	6.153	**0.034**	3.178	**0.003**
30–34	3.528	0.163	4.841	0.248	7.010	0.063	6.305	**0.018**	6.091	0.142	4.356	**0.011**
35–39	6.108	0.095	6.747	0.121	10.791	**0.020**	9.576	**0.001**	12.608	**0.008**	7.950	**0.002**
40–44	8.045	0.139	6.077	0.186	13.199	**0.041**	11.395	**0.000**	17.800	**0.001**	9.104	**0.012**
45–49	5.045	0.412	6.734	0.150	10.635	0.134	11.111	**0.001**	14.329	**0.009**	8.042	0.076
50–54	3.151	0.622	3.278	0.418	9.717	0.182	9.593	**0.018**	6.129	0.231	7.408	0.164
55–59	4.110	0.411	1.984	0.406	8.234	0.120	14.256	**0.001**	6.118	0.135	4.717	0.312
GDP growth rate (%)												
20–24	−0.506	**0.013**	−0.020	0.962	−0.564	**0.021**	0.456	0.237	0.610	0.182	−0.154	0.396
25–29	−1.758	**0.000**	−2.141	**0.026**	−2.049	**0.003**	0.608	0.334	0.173	0.865	−0.435	0.243
30–34	−2.581	**0.002**	−2.954	**0.033**	−3.183	**0.011**	1.219	0.191	0.607	0.669	−0.824	0.153
35–39	−2.784	**0.022**	−2.755	0.058	−3.213	**0.042**	1.232	0.246	−0.773	0.643	−1.014	0.261
40–44	−3.900	**0.030**	−2.401	0.116	−5.111	**0.018**	0.913	0.423	−2.088	0.267	−1.730	0.165
45–49	−3.662	0.074	−3.360	**0.046**	−4.681	**0.048**	0.549	0.651	−3.733	0.072	−2.084	0.174
50–54	−3.398	0.103	−1.471	0.285	−4.732	0.051	0.254	0.857	−1.069	0.533	−2.934	0.101
55–59	−2.393	0.159	−0.387	0.622	−3.585	**0.043**	−0.222	0.884	−2.575	0.074	−2.530	0.099

Note: Statistical significance value was marked in bold.

## Supplementary Materials

The following are available online at https://www.mdpi.com/1660-4601/16/11/2007/s1. Table S1: Suicidal mortality per 100,000 according to occupational group using direct standardization by 5 years age group; Table S2: Standardized Mortality Ratio and 95% Confidence Interval of Men; Table S3: Standardized Mortality Ratio and 95% Confidence Interval of Women.
