# Supplementary material for: Suicide Trends over Time by Occupation in Korea and Their Relationship to Economic Downturns
Source: Int J Environ Res Public Health. 2019 Jun 5;16(11):2007. doi: 10.3390/ijerph16112007 (PMC6604014; doi:10.3390/ijerph16112007)
Supplement: Supplementary file 1 [file ijerph-16-02007-s001.pdf]

Supplementary Table 1. Suicidal mortality per 100 thousand according to occupational group using direct standardization by 5 years age group

Men		1993	1994	1995	1996	1997	1998	1999	2000	2001	2002	2003	2004
	Manager	2.25	2.55	2.77	2.77	6.01	13.16	8.14	6.78	6.36	9.53	11.5	12.16
	Officer	12.07	10.24	23.14	26.98	19.74	29.3	21.67	17.31	19.09	22.05	25.55	27.19
	Service and trade	12.22	14.37	17.05	22.13	25.42	38.45	25.52	25.54	24.82	33.92	47.57	48.67
	Agricultural, fishery and forestry	54.24	47.89	53.27	57.83	53.26	79.01	72.96	69.61	68.07	87.49	87.36	75.1
	Skilled manual	12.83	13.82	15.54	17.85	18.99	22.65	14.79	10.39	10.33	13.63	14.76	13.75
	Unskilled manual	21.04	22.6	26.52	34.4	32.99	53.51	33.77	36.23	34.76	37.13	45.93	44.09
		2005	2006	2007	2008	2009	2010	2011	2012	2013	2014	2015	2016
	Manager	12.91	10.94	11.87	13.07	20.86	22.43	20.83	24.45	28.72	28.9	27.68	28.4
	Officer	25.38	24.84	24.65	26.15	36.28	38.63	41.5	28.46	27.02	25.66	21.22	19.35
	Service and trade	45	39.45	38.01	36.84	46.82	47.12	45.53	45.44	46.8	45.51	40.18	36.51
	Agricultural, fishery and forestry	71.29	58.21	60.56	53.22	57.89	62.81	69.68	53.18	61.9	58.29	49.65	43.26
	Skilled manual	12.54	9.75	8.71	11.91	13.38	12.36	13.71	14.55	16.49	15.1	15.29	14.34
	Unskilled manual	36.67	29.45	27.01	35.08	42.34	37.14	39.04	45.84	53.7	58.82	49.06	47.89
Women		1993	1994	1995	1996	1997	1998	1999	2000	2001	2002	2003	2004
	Manager	4.9	1.52	2.55	3.68	5.13	7.6	4.22	2.76	2.66	3.12	4.19	5.43
	Officer	10.3	6.77	8.4	8.68	4.22	6.21	5.38	5.24	3.45	4.56	10.11	7.17
	Service and trade	3.62	3.62	4.41	4.86	6.44	7.16	5.56	5.48	4.33	6.49	9.16	9.65
	Agricultural, fishery and forestry	17.02	17.64	18.81	21.16	14.13	21.49	21.34	21.79	24.16	23.39	40.85	26.62
	Skilled manual	3.42	3.68	2.66	2.04	2.68	2.28	1.81	1.66	2.22	3.75	4.36	2.28
	Unskilled manual	1.37	2.22	3	3.15	7.48	7.07	6.87	9.71	4.04	10.04	8.82	11.45
		2005	2006	2007	2008	2009	2010	2011	2012	2013	2014	2015	2016
	Manager	4.84	3.22	6.94	7.37	11.66	9.45	8.85	7.09	9.57	8.26	7.94	6.91
	Officer	11.11	8.74	12.38	14.02	15.32	12.2	13.02	11.16	8.35	9.51	7.39	7.53
	Service and trade	10.79	8.74	13.25	14.28	17.43	15.29	13.08	13.49	14.77	14.63	15.15	16.07
	Agricultural, fishery and forestry	22.58	17.62	12.37	13.86	12.73	16.16	14.62	13.76	10.63	15.11	13.86	10.61
	Skilled manual	2.31	1.32	4.08	5.39	5.47	3.42	2.92	5.01	3.81	5.83	5.71	4.45
	Unskilled manual	11.96	9.27	9.05	7.28	5.65	5.51	5.08	8.52	11.19	13.55	8.54	14.26

Supplementary Table 2. Standardized Mortality Ratio and 95% Confidence Interval of Men

Year	1993	1994	1995	1996	1997	1998	1999	2000
**Manager**	10 (8-13)	11 (8-14)	13 (10-16)	12 (10-15)	26 (22-30)	55 (49-61)	35 (30-40)	28 (24-33)
**Officer**	53 (44-64)	51 (42-61)	93 (81-107)	123 (109-138)	91 (79-104)	126 (113-140)	86 (76-98)	72 (63-83)
**Service and trade**	53 (46-61)	63 (55-72)	76 (67-85)	97 (87-107)	111 (100-122)	163 (151-177)	109 (98-120)	107 (97-118)
**Agricultural, fishery and forestry**	184 (168-201)	148 (134-164)	171 (156-188)	185 (169-203)	173 (157-191)	256 (235-278)	245 (224-267)	237 (216-259)
**Skilled manual**	58 (53-64)	61 (56-67)	68 (62-74)	78 (72-85)	83 (76-90)	97 (90-105)	64 (58-70)	44 (39-49)
**Unskilled manual**	78 (65-93)	88 (74-104)	101 (86-117)	128 (111-147)	129 (112-147)	212 (191-234)	133 (117-151)	144 (128-163)
	2001	2002	2003	2004	2005	2006	2007	2008
**Manager**	26 (22-30)	39 (34-44)	49 (43-55)	50 (45-56)	54 (48-60)	46 (41-52)	50 (45-56)	55 (50-61)
**Officer**	84 (74-94)	91 (81-102)	107 (96-118)	113 (103-125)	104 (95-115)	100 (91-111)	107 (98-118)	112 (102-123)
**Service and trade**	104 (94-115)	144 (132-157)	202 (188-217)	206 (191-220)	192 (178-206)	167 (154-180)	161 (148-173)	156 (144-168)
**Agricultural, fishery and forestry**	235 (214-258)	287 (263-313)	288 (264-315)	278 (253-305)	264 (239-290)	231 (208-257)	238 (213-265)	220 (195-247)
**Skilled manual**	45 (40-50)	58 (52-64)	63 (57-69)	59 (53-64)	53 (48-59)	42 (37-46)	37 (33-41)	51 (46-56)
**Unskilled manual**	143 (126-161)	149 (132-167)	183 (165-203)	181 (163-201)	152 (135-170)	122 (107-138)	111 (97-126)	143 (127-160)
	2009	2010	2011	2012	2013	2014	2015	2016
**Manager**	88 (81-95)	93 (86-101)	88 (80-95)	102 (95-111)	123 (114-132)	124 (116-133)	118 (110-127)	122 (113-130)
**Officer**	154 (143-166)	164 (153-177)	178 (166-191)	120 (110-130)	114 (105-124)	108 (99-117)	88 (80-96)	80 (73-88)
**Service and trade**	198 (185-211)	199 (186-212)	193 (181-207)	190 (178-203)	197 (185-210)	191 (179-204)	169 (158-180)	153 (143-164)
**Agricultural, fishery and forestry**	236 (210-265)	258 (230-289)	248 (220-279)	219 (192-248)	241 (212-273)	198 (171-228)	201 (173-232)	173 (147-202)
**Skilled manual**	56	52	58	57	65	59	60	56

	(51-61)	(47-57)	(53-63)	(52-62)	(60-71)	(54-64)	(56-65)	(51-60)
**Unskilled manual**	174<br>(157-192)	148<br>(133-165)	157<br>(142-174)	190<br>(173-208)	216<br>(198-235)	231<br>(213-249)	205<br>(188-222)	198<br>(182-215)

Supplementary Table 3. Standardized Mortality Ratio and 95% Confidence Interval of Women

**Year**	1993	1994	1995	1996	1997	1998	1999	2000
**Manager**	36<br>(24-52)	31<br>(20-46)	37<br>(25-53)	39<br>(27-55)	57<br>(43-75)	75<br>(59-95)	45<br>(32-60)	35<br>(24-48)
**Officer**	53<br>(37-74)	42<br>(28-61)	79<br>(59-103)	80<br>(61-104)	69<br>(51-90)	75<br>(58-96)	68<br>(52-87)	53<br>(39-70)
**Service and trade**	44<br>(34-57)	45<br>(35-58)	57<br>(45-72)	60<br>(48-75)	75<br>(62-91)	85<br>(71-101)	66<br>(55-80)	64<br>(53-77)
**Agricultural, fishery and forestry**	170<br>(141-203)	160<br>(132-192)	167<br>(137-200)	182<br>(151-218)	139<br>(111-172)	210<br>(174-252)	189<br>(154-231)	204<br>(165-248)
**Skilled manual**	43<br>(29-63)	50<br>(34-71)	32<br>(20-50)	26<br>(15-43)	34<br>(21-52)	28<br>(16-45)	20<br>(10-35)	20<br>(10-36)
**Unskilled manual**	20<br>(8-42)	29<br>(14-54)	38<br>(20-66)	29<br>(14-54)	73<br>(47-108)	84<br>(56-122)	66<br>(41-100)	101<br>(69-142)
	2001	2002	2003	2004	2005	2006	2007	2008
**Manager**	29<br>(20-41)	29<br>(20-40)	48<br>(36-61)	57<br>(44-71)	57<br>(45-72)	41<br>(31-53)	81<br>(68-97)	88<br>(74-104)
**Officer**	45<br>(33-61)	67<br>(52-85)	97<br>(79-117)	82<br>(66-101)	150<br>(129-175)	97<br>(80-116)	166<br>(144-191)	178<br>(156-203)
**Service and trade**	49<br>(40-61)	80<br>(67-94)	110<br>(95-127)	121<br>(104-138)	126<br>(109-144)	103<br>(89-120)	144<br>(127-163)	156<br>(139-175)
**Agricultural, fishery and forestry**	233<br>(190-282)	221<br>(179-271)	264<br>(216-320)	269<br>(219-327)	232<br>(184-289)	192<br>(148-247)	169<br>(126-223)	206<br>(156-266)
**Skilled manual**	25<br>(14-43)	41<br>(26-62)	43<br>(27-64)	26<br>(15-43)	28<br>(16-45)	15<br>(7-29)	43<br>(28-63)	52<br>(35-74)
**Unskilled manual**	43<br>(24-72)	73<br>(47-109)	97<br>(66-138)	110<br>(76-155)	108<br>(74-152)	58<br>(36-90)	92<br>(66-125)	58<br>(40-81)
	2009	2010	2011	2012	2013	2014	2015	2016
**Manager**	133<br>(116-152)	113<br>(98-130)	102<br>(88-117)	86<br>(73-101)	109<br>(94-124)	99<br>(86-114)	88<br>(76-102)	81<br>(69-94)
**Officer**	197<br>(174-222)	152<br>(133-174)	162<br>(142-185)	140<br>(121-160)	109<br>(93-128)	113<br>(97-132)	94<br>(79-110)	93<br>(79-109)
**Service and trade**	191<br>(172-211)	171<br>(154-190)	152<br>(136-170)	155<br>(138-173)	170<br>(153-189)	172<br>(154-191)	164<br>(147-183)	183<br>(165-203)
**Agricultural, fishery and forestry**	186<br>(138-246)	212<br>(158-278)	224<br>(167-295)	197<br>(141-267)	159<br>(108-228)	141<br>(90-210)	174<br>(115-254)	99<br>(54-166)
**Skilled manual**	48	33	29	48	42	61	60	44

	(32-69)	(20-51)	(17-46)	(33-69)	(27-61)	(43-84)	(43-83)	(29-64)
**Unskilled manual**	53<br>(37-74)	47<br>(33-66)	53<br>(37-73)	93<br>(71-119)	124<br>(97-156)	125<br>(97-158)	95<br>(71-125)	116<br>(89-149)
